# Robot-assisted Müllerian compartment resection for cervical cancer

**DOI:** 10.3389/fonc.2024.1466921

**Published:** 2024-10-15

**Authors:** Ya Li, Jing Na, Xinyou Wang, Shichao Han, Jun Wang

**Affiliations:** Obstetrics and Gynecology department, the second affiliated hospital of Dalian Medical University, Dalian, China

**Keywords:** cervical cancer, membrane anatomy, robot, menbrane bridge, Müllerian compartment

## Abstract

**Objective:**

Radical hysterectomy has been established as the standard treatment for early stage cervical cancers. Despite numerous efforts to standardize the technique for radical hysterectomy across varying extents of tumor invasion, success has been inconsistent. Total Müllerian Compartment Resection (TMCR), an ontogenetic compartment-based oncologic surgery initially developed for open procedures by Professor Höckel, offers a standardized approach applicable to all patients with locally confined tumors. This method holds promise for achieving thorough oncologic clearance while maintaining acceptable complication rates. Moreover, robotic-assisted surgery may further reduce morbidity compared to open surgery. In this context, we provide a detailed step-by-step description of robotically assisted Total Müllerian Compartment resection (R-TMCR) for cervical cancer and present feasibility data from a cohort of 20 patients.

**Subjects and methods:**

20 patients with stage IA1-IB2 cervical cancer, robot-assisted resection of the Müllerian embryonic compartment was undertaken. Key metrics such as operative duration, intraoperative blood loss, and postoperative complication rates were meticulously recorded and analyzed.

**Results:**

The duration of the surgery varied from 185 to 500 minutes, with intraoperative blood loss ranging between 5 mL and 300 mL. Postoperative hemoglobin levels dropped by -15 to 40 g/L from their preoperative values. Notably, there were no instances necessitating conversion to open surgery, and no intraoperative complications occurred. The rate of postoperative complications was 0%. Over the follow-up period, which averaged 18 months, there were no observed locoregional recurrences of cervical cancer, nor were there any deaths attributed to cervical cancer during this time.

**Conclusion:**

The application of robotic Müllerian compartment resection in the surgical treatment of cervical cancer is both safe and feasible. Utilizing robotic technology enables more precise and refined surgical outcomes. Combining embryonic compartment-based radical hysterectomy with the principles of membrane anatomy can standardize and optimize the surgical process, helping surgeons master radical hysterectomy more quickly and effectively.

## 1 Introduction

Extensive surgical resection is considered an effective method for controlling local recurrence in most solid malignant tumors. Achieving microscopically negative surgical margins (R0 resection) after tumor removal is crucial for preventing local recurrence. However, even with microscopic tumor-free status achieved through extensive resection, the local recurrence rate can be as high as 50% without adjuvant therapy (1, 2). Therefore, if preoperative or postoperative pathology indicates specific high-risk histological factors, neoadjuvant or adjuvant radiation or chemoradiation may reduce the likelihood of local recurrence following surgical resection. Nonetheless, this multimodal therapy may increase the risk of additional complications (2).

The embryonic compartment theory posits that the growth of malignant tumors is restricted within anatomical morphogenetic compartment derived from common primordia during embryonic development. These embryonic compartments are enveloped by their own membranes, allowing tumors within the compartment to freely invade. However, the presence of membranes surrounding the embryonic compartment restricts tumor invasion and spread outwardly (3). This theory was validated through studies on Drosophila (4), confirming the existence of embryonic compartments, as reviewed by Dahmann et al (5).

Some clinical data regarding the restricted spread of tumor cells within embryonic compartments have been gathered from studies on rectal carcinoma. The rectum, differentiated from the embryonic hindgut, includes the rectum and the enveloping mesorectum, together forming a distinct embryonic compartment (6). During the progression of rectal cancer, tumor cells spread by both continuous and discontinuous propagation but are confined within the embryonic compartment by the surrounding membrane (7).The validity of this theory can also be demonstrated in the distal part of the rectal compartment, which contains the internal anal sphincter. In contrast, the external anal sphincter belongs to a different embryonic compartment derived from the sacral somites. Only rectal carcinomas in very advanced stages of malignant progression can invade the external anal sphincter, thereby crossing into a different embryonic compartment (8).

Therefore, effective control of local tumor recurrence does not simply entail pursuing extensive resection but rather achieving complete resection of the embryonic compartment without damaging its outer membrane.

Subjects: Patients with stage IB1-IIA2 cervical cancer (FIGO 2018) and no evidence of lymph node metastasis as determined by magnetic resonance imaging (MRI) or computed tomography (CT).

## 2 Method

### 2.1 Surgical technique

All surgical procedures were performed under the guidance of membrane anatomy principles. By opening the connective tissue between the intraperitoneal embryonic compartments, known as menbrane bridge, we accessed the extraperitoneal membrane spaces between these embryonic compartments. Dissecting through these extraperitoneal spaces exposed the extraperitoneal connections between the embryonic compartments, also referred to as extraperitoneal membrane bridges. By severing these extraperitoneal membrane bridges, we achieved the separation of the embryonic compartments and isolation of the target compartment, allowing for the complete resection of the Müllerian compartment.

### 2.2 Statistical analysis

All data were analyzed using SPSS version 21.0. Due to the small sample size and the exploratory nature of this study, we performed only descriptive statistical analyses on the collected data.

### 2.3 Surgical steps

#### 2.3.1 Uterine suspension

**Figure 1:**
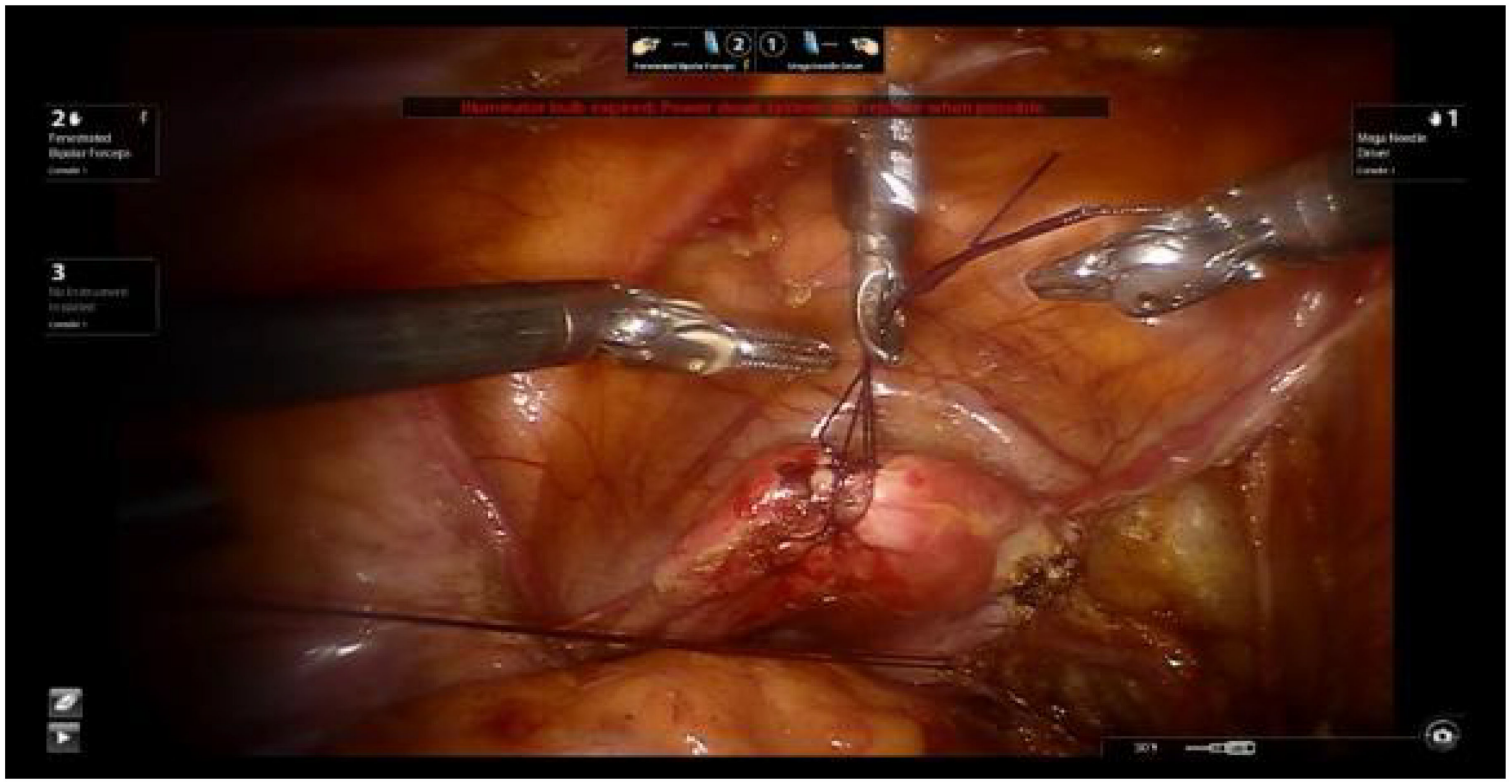
Shows uterine suspension.

Using 1-0 absorbable sutures, two ‘8’ stitches are placed at the uterine fundus. The uterus is manipulated using a laparoscopic needle holder to grasp the uterine sutures and move the uterus, replacing the uterine manipulator. This method avoids compressing the cervical tumor and prevents tumor spillage into the vagina, thereby reducing the risk of tumor dissemination (**Figure 1**).

#### 2.3.2 Lateral parametrium

The lateral parametrium is the mesometrial outlet of the Müllerian embryonic compartment, containing the uterine artery, superficial uterine vein, and deep uterine veins.

**Figure 2:**
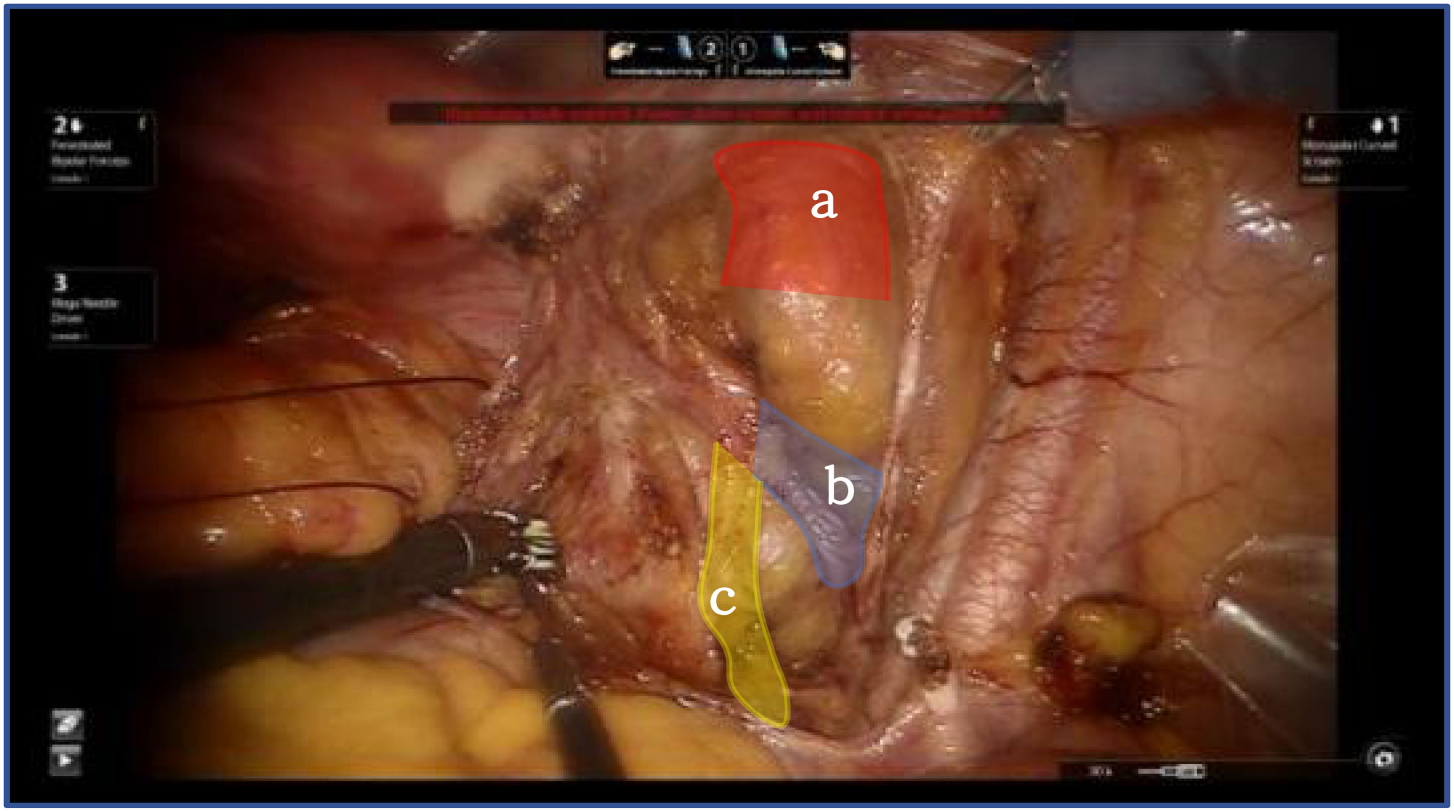
Shows the lateral parametrium, red shows bladder of the urogenital embryonic compartment "a", blue show mesometrial outflow of the Müllerian embryonic compartment "b", yellow ureter of the ureteric bud embryonic compartment "c".

Thorough dissection of the lateral retroperitoneal space reveals the paravesical space, Latzko’s pararectal space, and Okabayashi’s pararectal space. The lateral parametrium is located between the paravesical space and Latzko’s pararectal space. This dissection epitomizes the principles of membrane anatomy surgery, emphasizing the segregation of embryonic compartments. The delineation of the paravesical space pertains to the separation between the bladder of the urogenital embryonic compartment and the mesometrial outflow of the Müllerian embryonic compartment. Conversely, the delineation of Latzko’s pararectal space pertains to the separation between the mesometrial outflow of the Müllerian embryonic compartment and the ureter of the ureteric bud embryonic compartment (**Figure 2**).

#### 2.3.3 Dorsal parametrium

**Figure 3:**
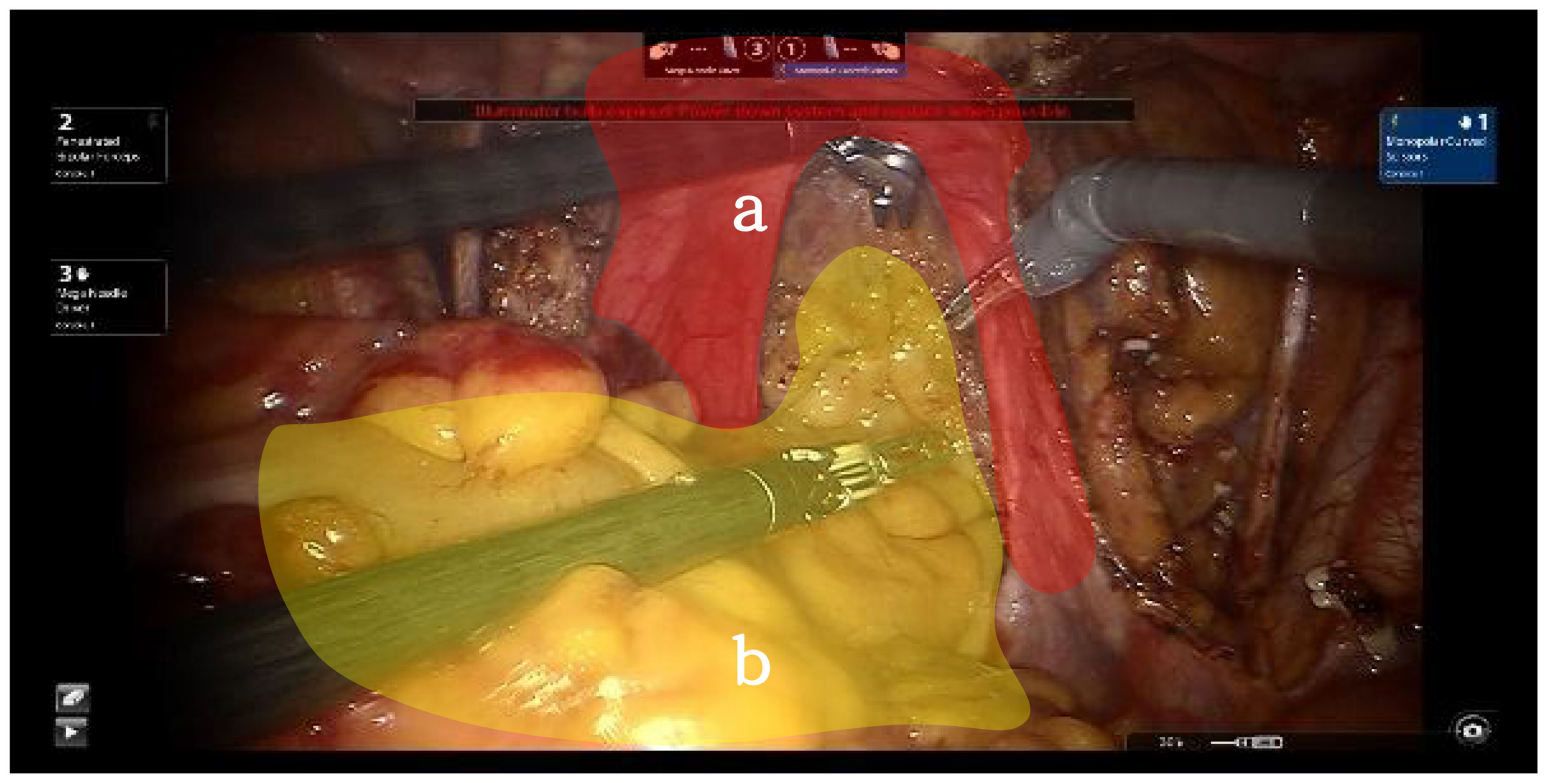
Red shows Müllerian embryonic compartment "a", yellow shows hindgut embryonic compartment "b".

The dorsal parametrium is the supporting structure of the Müllerian embryonic compartment, known as the sacrouterine ligament. The exposure of the dorsal parametrium involves separating the rectum of the hindgut embryonic compartment from the sacrouterine ligament and the vagina of the Müllerian embryonic compartment. This dissection only involves the separation of two embryonic compartments; thus, transection of the membrane bridge (i.e., the vaginal peritoneal reflection) between the two compartments allows access to the inter-compartmental space, achieving a bloodless and complete separation of the two compartments (9) (**Figure 3**).

#### 2.3.4 Ventral parametrium

**Figure 4:**
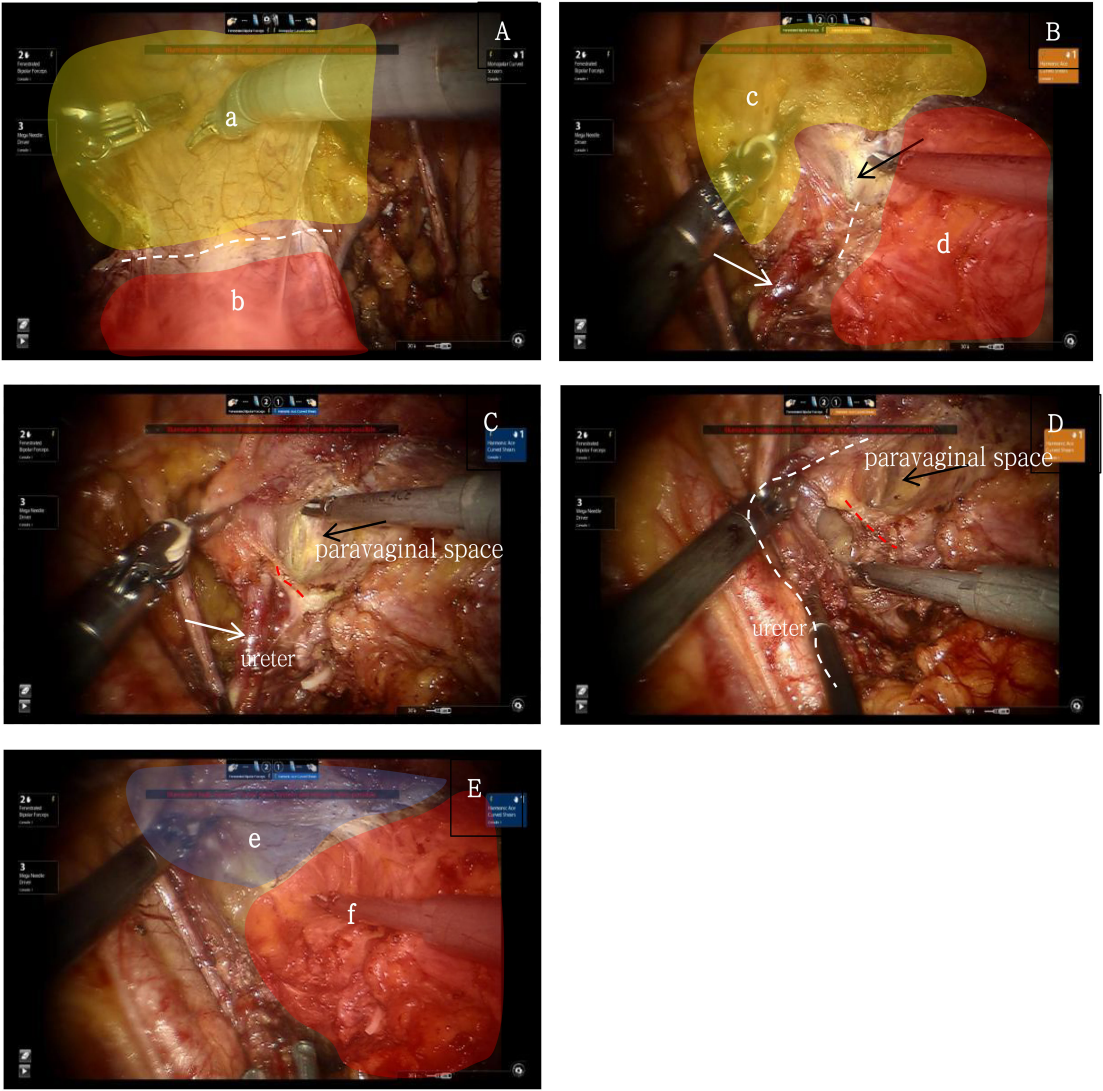
**(A)** yellow shows the urogenital embryonic compartment "a", red shows the Müllerian embryonic compartment "b", white dash line shows the membrane bridge(i.e., the vesicouterine peritoneal fold) between the two embryonic compartments; **(B)** yellow shows the urogenital embryonic compartment "c", red shows the Müllerian embryonic compartment "d", white dash line shows the membrane bridge(i.e., the vesicocervical ligament) between the two embryonic compartments, white arrow shows the ureter, black arrow shows the “Fourth Space”; **(C)** red dash line shows the membrane bridge between the ureteric bud embryonic compartment and the Müllerian embryonic compartment, white arrows show the ureter, black arrow shows the paravaginal space; **(D)** red dash line shows the membrane bridge(i.e., the vesicovaginal ligament) between the urogenital embryonic compartment and the Müllerian embryonic compartment, white dash line shows the ureter, black arrow shows the paravaginal space; **(E)** shows the completely separation of the urogenital embryonic compartment "e" and the Müllerian embryonic compartment "f".

The ventral parametrium involves the separation of the parametrium and paracolpium of the Müllerian embryonic compartment, the ureter of the ureteric bud embryonic compartment, and the bladder of the urogenital embryonic compartment, making it more challenging. First, transection of the membrane bridge between the Müllerian embryonic compartment and the urogenital embryonic compartment (i.e., the vesicouterine peritoneal fold) allows access to the inter-compartmental spaces of these two compartments, namely the vesicocervical space and the vesicovaginal space. By continuing to expand the inter-compartmental spaces laterally, the fourth space is exposed, revealing the extraperitoneal membrane bridge between the urogenital embryonic compartment and the Müllerian embryonic compartment (i.e., the vesicocervical ligament). Transection of this membrane bridge and further lateral expansion of the inter-compartmental spaces between the urogenital and Müllerian embryonic compartments will reveal the paravaginal space. Adequate exposure of the paravaginal space will show the extraperitoneal membrane bridge between the urogenital embryonic compartment and the Müllerian embryonic compartment in the paravaginal connective tissue (i.e., the vesicovaginal ligament). Transection of this membrane bridge completes the separation of the ventral parametrium, involving the urogenital embryonic compartment, the ureteric bud embryonic compartment, and the Müllerian embryonic compartment (10) (**Figure 4**).

#### 2.3.5 Complete resection of the Müllerian embryonic compartment

**Figure 5:**
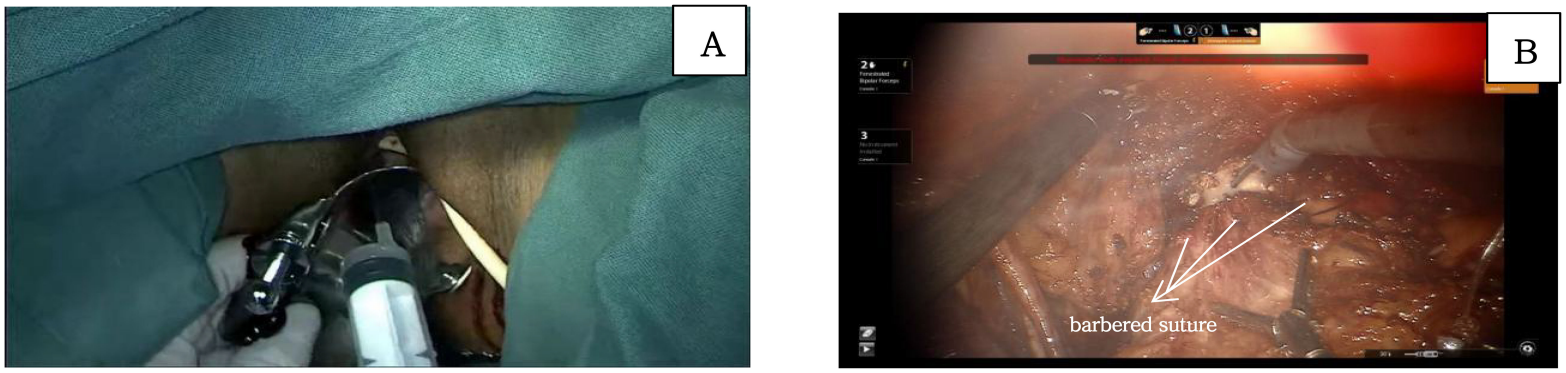
**(A)** shows the flushing of vaginal with sterile distilled water; **(B)** shows the incision of the vaginal and the closure of vaginal with barbered suture.

At the level of the levator ani fascia, the paravaginal connective tissue is transected perpendicular to the axis of the vagina. The vaginal cuff is then closed using a barbed suture, Ensure that the sutures do not penetrate through the vaginal wall, achieving a tight closure to prevent tumor spillage. Prior to vaginal incision, the vaginal cavity is repeatedly irrigated with sterile distilled water heated to 42°C. This precaution is taken to prevent the shedding of cervical tumor cells into the vaginal cavity, thereby minimizing the risk of tumor exposure and dissemination (**Figure 5**).

## 3 Results

A total of 20 patients diagnosed with cervical cancer underwent surgical treatment. All patients received an embryonic compartment-based hysterectomy. Due to positive pelvic lymph nodes identified in the postoperative pathological examination, 2 patients received adjuvant chemotherapy. The mean age of the patients was 49.35 years (range: 28-68 years). The mean body mass index (BMI) was 24.83 kg/m² (range: 19.43-32.38 kg/m²).

According to the 2018 FIGO staging system, there were 5 cases of stage IA1, 10 cases of stage IB1, 2 cases of stage IB2, and 1 case of stage IIA1. Additionally, 2 cases initially assessed clinically and radiologically as stage IB1 were confirmed as stage IIIC1p postoperatively. Among the cohort of 20 cases, squamous cell carcinoma predominated with a diagnosis in 18 patients (90%), while adenocarcinoma was less frequent, identified in 2 cases (10%). Postoperative pathological examination disclosed lymph node involvement in 2 cases (10%) and lymphovascular invasion (LVSI +) in 4 tumors (20%). All surgical interventions proceeded as planned, with no conversions to open surgery necessitated by complications or technical challenges. Microscopic evaluation confirmed complete (R0) tumor resection in every case. The mean number of pelvic lymph nodes harvested during lymphadenectomy was 20.7. There were no intraoperative and postoperative complications. With respect to blood loss, hemoglobin levels were determined pre and postoperatively (on the first day). Mean decrease of hemoglobin concentration was determined to be 11.2 g/l in RTMCR (-15 to 40 g/l), Postoperative follow-up revealed an increase in hemoglobin levels in 3 cases compared to preoperative levels, primarily due to the no bleeding during surgery. Intraoperative and postoperative blood transfusions were not required for any of the surgical patients. Mean follow-up of the patients was 18 months (range 6 to 36 months).

## 4 Discussion

The concept of compartment-based resection, guided by membrane anatomy and embryonic development theory, provides valuable insights into the radical surgical resection of local tumors. Instead of evaluating the width of tumor margins relative to the specimen edges after compartmental resection, the focus shifts to ensuring the complete resection of the embryonic compartment as the standard criterion. This approach maximizes the removal of the target compartment, achieving optimal local control without the need for adjuvant radiation while minimizing treatment-related complications and avoiding functional damage to adjacent embryonic compartment. Compartment-based resection can be adapted to wide resection within the embryonic compartment depending on tumor staging. For locally advanced cancers, supra-compartmental or multi-compartmental resection may be considered as treatment options. This perspective has been validated in clinical studies of cervical cancer (11, 12), vulvar cancer (13), vaginal cancer (14), and rectal cancer (8). The data from these studies highlight the significant potential of compartment-based resection to improve local tumor control, reduce treatment-related complications, and enhance overall survival. A study conducted on 4000 patients found that total mesorectal excision resulted in a 20% increase in overall survival compared to traditional surgery (7). Similar survival benefits were observed in a single-center trial for cervical cancer patients undergoing total mesometrial resection by Professor Höckel (12).

Through our clinical experience, we’ve observed that the principle of embryonic compartment surgery, as elucidated by M. Höckel (15), in total mesometrial resection (TMMR), can also be effectively applied through advanced laparoscopic techniques and robotic assistance (rTMMR). The enhanced visualization provided by robotic-assisted surgery facilitates more precise tissue dissection. Utilizing the magnified and clear surgical field provided by robotics, we can precisely identify critical structures like membrane boundaries, bridges, and inter-compartmental spaces. This precision enables us to achieve meticulous resection of compartment-associated tissue while preserving adjacent structures.

Robotic surgery has been thoroughly investigated for its safety and effectiveness in treating cervical cancer (16, 17). However, the outcomes of the (18) have contradicted earlier findings and raised concerns regarding the safety of minimally invasive procedures (16). Subsequent retrospective studies, both corroborating and disputing the findings of the LACC trial, have been published (19–23). Nonetheless, these studies have not revealed any substantial differences in oncological outcomes between the two surgical approaches. In this context, with the continuous advancement of no-tumor microinvasive surgical procedures, robotic surgery, offering excellent three-dimensional visualization and flexible mechanical arms, has demonstrated significant advantages (24). In recent years, Professor Kimmig and Professor Höckel have both performed robot-assisted TMMR (25), and the results have confirmed its safety and efficacy. Following the principle of no-tumor surgery and utilizing refined surgical techniques, this study conducted robotic Müllerian compartment resection. Based on clinical observations, the application of robotic-assisted embryonic compartment-based hysterectomy appears to be feasible and safe. However, with an average follow-up time of 18 months and a total of only 20 cases, the study’s sample size is limited and the follow-up time is relatively short.

Patients were closely monitored for perioperative complications. Intraoperative complications included bleeding, organ injury, and anesthesia-related complications. Postoperative complications included infections (such as surgical site infections and pelvic infections), thromboembolism (deep vein thrombosis and pulmonary embolism), urinary complications (including urinary retention, bladder fistula, and ureteral injury), and bowel obstruction (delayed postoperative bowel function recovery or mechanical bowel obstruction). In this study, none of the 20 patients experienced any of the aforementioned complications. Based on clinical observations, the application of robotic-assisted embryonic compartment-based hysterectomy appears to be feasible and safe.

In this study, with an average follow-up period of 18 months and a total of 20 cases, the sample size is limited and the follow-up duration is relatively short. However, since intraoperative and postoperative complications do not significantly increase with longer follow-up, this study demonstrates clear superiority compared to previous studies (26), particularly in the management of urinary system injuries, which are both more frequent and challenging to handle. Notably, there were no instances of urinary tract injury in this study, highlighting a significant advantage. This also indicates that embryonic compartment-based hysterectomy has unique benefits in managing the ventral parametrium, especially in the dissection of membrane bridges between different embryonic compartments and the layered management of the periureteral membrane bridge, making it worthy of further standardized promotion.

Although the follow-up period was relatively short, with a median duration of only 18 months, there were no cases of recurrence. In comparison, studies on traditional surgical methods report a recurrence rate of 3-4% within the first year (27), which suggests that this study has demonstrated preliminary oncological advantages. Since it is widely believed that recurrences within the first year are primarily due to incomplete surgical resection, embryologically-based Müllerian compartment hysterectomy shows a distinct advantage in ensuring thorough removal. The concept of membrane anatomy goes beyond just organ removal; it involves the complete excision of the histological boundaries defined by the organ’s developmental origins. This highlights the importance of improvements in surgical concepts and techniques in enhancing oncological outcomes. Despite this limitation, no instances of local tumor recurrence were observed throughout the entire follow-up period. Therefore, it is reasonable to hypothesize that R-TMCR is safe and feasible for local tumor control. It is imperative to continue accumulating more cases and extending the follow-up period to further substantiate this observation.

In this study, 20 patients successfully underwent embryonic compartment radical hysterectomy guided by the principles of menbrane anatomy. Although the sample size is small and the follow-up period is relatively short, preliminary results indicate advantages in rapid recovery and favorable oncological outcomes. Particularly under the guidance of tumor-free principles, every detail of the procedure adhered strictly to tumor-free principles. The techniques for maintaining a tumor-free environment are simple, standardized, and easy to learn, which enhances their potential for broader adoption and minimizes the risk of tumor recurrence.

Pelvic lymphadenectomy is a crucial component of cervical cancer surgery. In this study, embryonic compartmental resection was performed in conjunction with traditional pelvic lymphadenectomy (which involved an average of 20.7 lymph nodes being removed). This approach differs from the more extensive and invasive therapeutic lymphadenectomy utilized in Professor Höckel’s Total Mesometrial Resection (TMMR) procedures (28). The considerations for this decision include the following:

Adherence to Clinical Guidelines (29): Conventional lymphadenectomy aligns with the recommendations of current authoritative guidelines such as those from the NCCN. Postoperative pathological examination provides critical staging information for positive lymph nodes and supports subsequent adjuvant therapy.

Challenges of Tumor Removal: Once lymph node metastases occur, from the perspective of “menbrane anatomy” originating from embryonic development, the surgery may exceed the excision limits of the paramesonephric duct embryonic unit (30). Even when performing therapeutic lymphadenectomy, operations confined to the embryonic unit may struggle to ensure complete tumor removal and carry the potential risk of distant metastases; thus, postoperative adjuvant radiotherapy and chemotherapy become particularly vital. Although Hocker’s study reported favorable oncological outcomes in early cervical cancer cases, experimental research in locally advanced patients, especially those not requiring radiotherapy and chemotherapy, did not demonstrate significant advantages (31). This is one of the primary reasons we opted not to perform therapeutic lymphadenectomy.

Clear Focus of the Study: The aim of the current study is to investigate the impact of a new technique for embryonic compartment-type hysterectomy on perioperative complications and short-term oncological outcomes, particularly concerning local recurrence. Concurrently performing therapeutic lymphadenectomy might hinder the direct comparison with traditional surgical methods, complicating the assessment of how variations in surgical techniques affect final outcomes.

Management of Positive and Negative Lymph Node Cases: According to the current FIGO 2018 guidelines (32), for preoperatively suspected positive lymph nodes revealed through imaging, it is recommended to perform lymph node staging surgery or concurrent radiotherapy and chemotherapy, rather than extensive hysterectomy. Therefore, such cases are not within the scope of this study. Compared to Hocker’s research (33), which was based on FIGO 2009 staging without considering lymph node metastasis in the staging process, more patients with lymph node metastasis may have been included (34), which decreases the comparability of the data.

Consideration of Surgical Risks: The extent of therapeutic lymphadenectomy is considerably broader and significantly increases the risks of bleeding and injury. Even with favorable oncological outcomes, experienced gynecological oncologists face considerable challenges when performing such procedures, which may hinder the promotion and implementation of these techniques in standard clinical practice.

We hope that this clarification aids in understanding the considerations and choices made in the design of our study. Further research with larger sample sizes and long-term follow-up is essential to address the limitations of this current study.

From the perspective of membrane anatomy, extensive resection surgery reveals that traditional methods do not achieve complete embryonic compartment resection. True complete embryonic compartment resection requires precise sharp dissection and cutting to preserve the membrane structure of the embryonic compartment, thereby ensuring containment of the tumor within the compartment. In contrast, traditional extensive resection emphasizes gross removal of the embryonic compartment, often involving blunt dissection and cutting methods. This approach may result in damage to adjacent embryonic compartments and compromise the integrity of the target compartment, potentially leading to tumor spillage.

## 5 Conclusion

From the perspective of membrane anatomy, extensive resection surgery reveals that traditional extensive resection does not achieve complete embryonic compartment resection. True complete embryonic compartment resection requires precise sharp dissection and cutting to preserve the membrane structure of the embryonic compartment, thereby ensuring containment of the tumor within the compartment. In contrast, traditional extensive resection emphasizes gross removal of the embryonic compartment, often involving blunt dissection and cutting methods. This approach may result in damage to adjacent embryonic compartments and compromise the integrity of the target compartment, potentially leading to tumor spillage.

The integration of robotic technology into radical hysterectomy surgeries offers the potential for enhanced surgical precision and finesse. By embracing meticulous surgical methodologies, it becomes possible to standardize and refine procedures, thereby streamlining the learning curve.

## Data Availability

The original contributions presented in the study are included in the article/**Supplementary Material**. Further inquiries can be directed to the corresponding author/s.
